# Genome sequence and evolution of *Betula platyphylla*

**DOI:** 10.1038/s41438-021-00481-7

**Published:** 2021-02-11

**Authors:** Su Chen, Yucheng Wang, Lili Yu, Tao Zheng, Sui Wang, Zhen Yue, Jing Jiang, Sapna Kumari, Chunfang Zheng, Haibao Tang, Jun Li, Yuqi Li, Jiongjiong Chen, Wenbo Zhang, Hanhui Kuang, Jon S. Robertson, Patrick X. Zhao, Huiyu Li, Shengqiang Shu, Yordan S. Yordanov, Haijiao Huang, David M. Goodstein, Ying Gai, Qi Qi, JiuMeng Min, ChunYan Xu, SongBo Wang, Guan-Zheng Qu, Andrew H. Paterson, David Sankoff, Hairong Wei, Guifeng Liu, Chuanping Yang

**Affiliations:** 1 State Key Laboratory of Tree Genetics and Breeding (Northeast Forestry University), Harbin, China; 2 BGI-Qingdao, BGI-Shenzhen, Qingdao, China; 3 BGI-Tech, BGI-Shenzhen, Shenzhen, China; 4 College of Forest Resources and Environmental Science, Institute of Computing and Cybersystems, Michigan Technological University, Houghton, MI USA; 5 Department of Mathematics and Statistics, University of Ottawa, Ottawa, ON Canada; 6 Center for Genomics and Biotechnology, Fujian Agriculture and Forestry University, Fuzhou, Fujian Province China; 7 School of Plant Sciences, University of Arizona, Tucson, AZ USA; 8 Noble Research Institute, 2510 Sam Noble Parkway, Ardmore, OK USA; 9 Department of Vegetable Crops, College of Horticulture and Forestry, Huazhong Agricultural University, Wuhan, P.R. China; 10 Plant Genome Mapping Laboratory, University of Georgia, Athens, Georgia; 11 US Department of Energy Joint Genome Institute, Walnut Creek, CA USA; 12 Department of Biological Sciences, Eastern Illinois University, Charleston, IL USA; 13 College of Biological Sciences and Biotechnology, Beijing Forestry University, Beijing, P. R. China

**Keywords:** Genomics, Plant breeding

## Abstract

Betula L. (birch) is a pioneer hardwood tree species with ecological, economic, and evolutionary importance in the Northern Hemisphere. We sequenced the *Betula platyphylla* genome and assembled the sequences into 14 chromosomes. The Betula genome lacks evidence of recent whole-genome duplication and has the same paleoploidy level as *Vitis vinifera* and *Prunus mume*. Phylogenetic analysis of lignin pathway genes coupled with tissue-specific expression patterns provided clues for understanding the formation of higher ratios of syringyl to guaiacyl lignin observed in *Betula* species. Our transcriptome analysis of leaf tissues under a time-series cold stress experiment revealed the presence of the MEKK1–MKK2–MPK4 cascade and six additional mitogen-activated protein kinases that can be linked to a gene regulatory network involving many transcription factors and cold tolerance genes. Our genomic and transcriptome analyses provide insight into the structures, features, and evolution of the *B. platyphylla* genome. The chromosome-level genome and gene resources of *B. platyphylla* obtained in this study will facilitate the identification of important and essential genes governing important traits of trees and genetic improvement of *B. platyphylla*.

## Introduction

*Betula platyphylla* is a medium-sized, broad-leaved, deciduous hardwood tree species of the genus *Betula* (Betulaceae). The genus *Betula* consists of 30–60 taxa^1^; 20 are native to northern Europe and Asia and 14 are native to North America. *Betula* species constitute 10% of China’s northeastern vegetation area and 13% of Russian vegetation, and more than 50% of vegetation areas in Canada, Alaska, the midwestern region, and the northeastern United States. Similar to most other *Betula* species, *B. platyphylla* is distributed across a wide range of geographical areas in the Northern Hemisphere, particularly in the Far East, Russia, and the northeastern regions of China and Japan (Fig. S1). *B. platyphylla* can emerge and colonize open ground in grasslands and on roadsides to increase and diversify its natural beauty in various landscapes. A *B. platyphylla* tree can grow to 20 m at a fast rate. It is in flower from April to May, and the seeds ripen in September. The species is monoecious, and individual flowers are either male or female, but both sexes can be found on the same plant. It is pollinated by wind.

*B. platyphylla* has a trunk with bright white bark, a pyramidal or oval crown form with a central leader, and thin, spreading, slightly pendant branches. It can be planted to produce natural and beautiful landscapes in all seasons. It sprouts early in spring and produces beautiful leaves and dangling flower clusters. In summer, the graceful pyramid- or oval-shaped crowns are draped with catkins and dense dark green foliage that can rustle under breezy conditions. In autumn, its leaves turn into a golden yellow color. When mixed with other species, such as maple, birch trees can generate visual impacts and multicolored landscapes. The primary ornamental feature of birch trees is their lovely creamy white bark with black fissures and branch marks, especially in the winter. The smooth white bark is extremely showy and adds significant winter interest. The *Betula platyphylla* cultivar Dakota pinnacle “fargo” (rfm-19), which has a narrow columnar to narrowly pyramidal growth habit and dense dark green foliage that turns golden yellow in the fall, has been selected and widely planted in some arboretum gardens and the northern plains in the USA^2^. *Betula platyphylla* var. *japonica* grows more frequently in grasslands that decorate temperate and boreal landscapes^3^. It is worth mentioning that there are many varieties of birch, such as Snow Queen birch (*B. utilis*), Canoe birch (*B. papyrifera*), Cherry birch (*B. lenta*), and River birch (*B. nigra*), which have different bark and crown features for meeting various decoration purposes. At least seven dwarf birch tree species, which include *B. nigra* “little king” and *B. nana*, can be planted in yards and gardens for greening and decoration. In addition to serving as landscape trees, wood from *Betula* species, including *B. platyphylla*, has been used since prehistoric times, primarily for building, crafting, and writing materials and secondarily for medication and cosmetics. Currently, *Betula* wood is used principally for lumber, veneer, pulpwood, fuel, and plywood. *Betula* bark extract exhibits immunomodulatory, anti-inflammatory, and antioxidant activities, and its pharmacological applications are intensively exploited^4^. The exceptional cold tolerance of *B. platyphylla* allows it to thrive in various landscapes in temperate and even boreal regions^5^.

Knowledge of the genomic sequence of *B. platyphylla* and its evolution is essential for improving its greening and landscape values, wood properties, and medicinal uses. Both conventional and molecular breeding strategies have been used in the last 30 years, including provenance tests, elite tree selection, cross-hybridization, marker-assisted selection, and genetic transformation^6^. However, without a reference genome assembly, these breeding strategies have reached an impasse. Here, we report the whole-genome sequencing and assembly of the 14 chromosomes (2*n* = 2x = 28) of an elite *B. platyphylla* tree. The genome sequence and gene resources we generated will be instrumental for evolution studies and genetic improvement of *Betula* species.

## Results and discussion

### Genome assembly

**Fig. 1:**
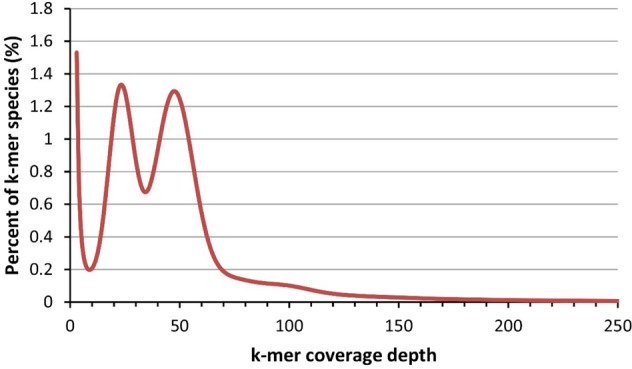
17-mer distribution of the Illumina reads

We first investigated the genomic features of *B. platyphylla* using a 17-mer distribution analysis of the Illumina data. A total of 26,432,968,648 17-mers were found in the Illumina sequencing data, and the 17-mer depth was 59. The 17-mer statistics suggested that the size of the *B. platyphylla* genome is approximately 441 Mb. In addition, the bimodal distribution of the 17-mer indicated that the *B. platyphylla* genome is highly heterozygous (Fig. 1). We assembled the PacBio reads into contigs using pb-assembly^7^ and wtdbg2 (ref. ^8^). The resulting contigs were merged using quickmerge^9^ and improved using FinisherSC^10^. The merged contigs were polished by Racon^11^ and Pilon^12^ with the PacBio and Illumina reads, respectively. The organelle genomes were removed by mapping the contigs to the birch chloroplast and mitochondrial genomes using blastn^13^. Finally, we obtained a 430 Mb assembly with a contig N50 length of 751 kb for the *B. platyphylla* genome (Table 1). The PacBio reads were mapped to the final genome assembly using minimap2 (ref. ^14^) to assess the genome quality. A total of 95.7% PacBio reads were successfully mapped to the genome assembly. Genome completeness was also assessed using BUSCO^15^. The BUSCO assessment results indicated that 95.5% of the BUSCO (94.3% complete and 1.2% fragmented) genes were found in the *B. platyphylla* genome assembly. The final contigs were further anchored to the birch chromosomes using a genetic map. The genetic map was constructed by specific-locus amplified fragment sequencing (SLAF-seq)^16^ technology using 250 F1 seedlings from the cross of *Betula platyphylla* Suk and *Betula pendula* var. *carelica*. The genetic map contained a total of 7900 markers. The total length and average distance of the genetic map were 1270.79 and 0.16 cM, respectively. Detailed information on the genetic map is listed in Table S1. We mapped the genetic markers to contigs using BWA-MEM^17^ and used ALLMAPS^18^ to anchor and orient the contigs to the genetic map. Finally, 91.3% of the contigs were anchored to the linkage group, of which 85.3% were oriented (Table 2).

**Table 1:** Statistics of the *B. platyphylla* genome assembly

Contig statistics	Length (bp)	Number
N10	2,676,567	13
N20	2,038,151	31
N30	1,307,637	59
N40	959,954	99
N50	751,259	150
N60	556,834	217
N70	390,922	309
N80	237,210	448
N90	114,006	714
Max. length	5,710,464	
Length ≥ 2000 bp		1550
Read number		1550
Total length	430,495,852

**Table 2:** Summary of the consensus map

Name	Anchored	Oriented	Unplaced
Markers (unique)	8317	7960	147
Markers per Mb	21.2	21.7	3.9
N50 contig	149	148	0
Contigs	939	684	601
Contigs with 1 marker	215	0	36
Contigs with 2 markers	126	108	26
Contigs with 3 markers	87	76	6
Contigs with ≥4 markers	511	500	7
Total bases	392,920,638 (91.3%)	367,137,491 (85.3%)	37,494,613 (8.7%)

After establishment of the genome assembly, we mapped the NGS reads to the assembly to evaluate the heterozygosity of *B. platyphylla*. We identified 3,756,661 single-nucleotide polymorphisms (SNPs) and 813,524 indels by using GATK (Genome Analysis Toolkit). The heterozygosity of the *B. platyphylla* genome was 1.20% according to the identified SNPs and indels. In addition, we also assessed the redundant sequences from highly differentiated haplotypes by investigating the sequencing coverage of the final assembly. We mapped approximately 36 coverages of the sequencing reads (NGS reads) to the genome assembly and found that most of the genome was covered 36 times by the sequencing reads. The results indicated that the final genome assembly contained minimal redundant sequences from the haplotype. However, a distinct peak was found at 72 times the sequencing coverage, which indicated that some repetitive sequences collapsed in the genome assembly (Fig. S2).

### Identification of repetitive sequences in the *B. platyphylla* genome

We found that the *Betula* genome contains a 183.5 Mb (43.0%) TE sequence (Table S2), which is comparable to that in *P. trichocarpa* (~44.2%)^19^, *Vitis vinifera* (~41.4%)^20^, and *Prunus mume* (~45.0%)^21^ but much higher than that in *Arabidopsis thaliana* (~18.5%)^22^. The predominant TEs in the *B. platyphylla* genome are Class I TEs, which account for 30.25% of the genome. The Class I TEs are composed of long terminal repeat (LTR) and non-LTR. The LTR TEs include Gypsy, Copia, and other unrepresentative types, which account for 9.23%, 9.11%, and 5.31% of the genome, respectively. The non-LTR TEs include long interspersed nuclear elements and small interspersed nuclear elements, which account for 6.56% and 0.03% of the genome, respectively. Class II DNA transposons constitute 8.29% of the genome, including 5.31% DNA transposons and 1.0% helitron transposons. Additionally, the *B. platyphylla* genome harbors 2.51 Mb (0.57%) simple sequence repeats, 15.52 Mb (3.52%) unclassified TEs, and 5.88% of other types of TEs.

Miniature inverted-repeat transposable elements (MITEs) preferably insert into proximal gene regions^23,24^ and therefore play critical roles in the functional evolution of genes. The *B. platyphylla* genome contains 155,721 MITEs from 126 families with a total length of 29.6 Mb, accounting for 6.7% of the genome. This percentage is much higher than that in *P. trichocarpa* (1.8%) and *Arabidopsis* (0.71%). Out of the 41 species that were genome-sequenced^25^, only *Oryza sativa* (9.98%) and *Medicago truncatula* (8.21%) had a greater percentage of MITEs. We classified these 126 MITE families into four superfamilies, CACTA, PIF/Harbinger, hAT, and Mutator (Table S3). The largest superfamily, Mutator, consists of 97,771 elements. Although the Tc1/Mariner superfamily is present in nine angiosperm genomes, including *P. trichocarpa*^26^, this superfamily is absent in the *B. platyphylla* genome. Approximately 88.5% of MITEs had lengths between 75 and 375 bp, and their AT contents varied from 65 to 90% (Fig. S3). We found that in the *B. platyphylla* genome, MITEs preferentially inserted into the 5′ and 3′ flanking regions of genes; 46.7% and 40.9% of MITEs were found in the 5 kb regions upstream and downstream of genes, respectively. Only 12.4% of MITEs were inserted into the coding regions (3472 bp) (Fig. S4). Interestingly, two regions, −250 to −750 bp upstream of start codons and 500 to 750 bp downstream of stop codons, harbored more MITEs than any other region. We extracted genes containing MITE elements (750 bp upstream regions to 750 downstream regions). These genes were then subjected to pairwise comparisons. We calculated the *Ks* distribution for the gene pairs. The results revealed an obvious peak at a small value of *Ks* (approximately 0.3), which indicated that MITE elements in *B. platyphylla* may contribute to genomic evolution to an extent (Fig. S5).

### Gene prediction and annotation

We identified 31,253 protein-coding genes in the *B. platyphylla* genome. Of these 31,253 genes, 24,817, 20,831, and 25,860 have Arabidopsis, SwissProt, and TrEMBL homologs, respectively. We also independently assembled RNA-seq data sets from different tissues, including leaves, apical meristems, roots, xylem, leaf veins, flowers, and entire plants, from tissue culture into transcripts, to validate the gene models. The assembled transcripts were mapped to the genome using minimap2 (ref. ^14^). In total, 24,046 of the annotated genomic loci were covered by the assembled transcripts. In addition to the protein-coding genes, a total of 396 miRNAs, 512 tRNAs, 265 rRNAs, and 331 small nuclear RNAs (snRNAs) were annotated in the *B. platyphylla* genome.

### Genome evolution

**Fig. 2:**
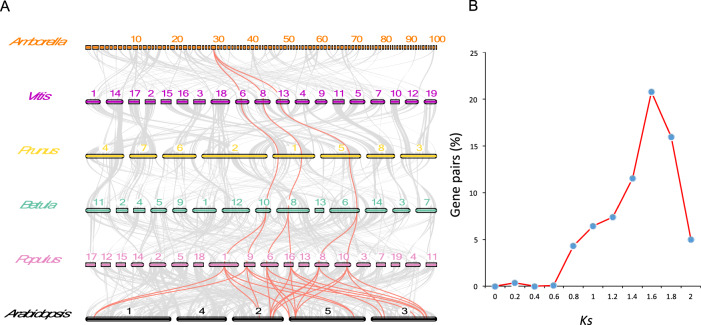
*Betula platyphylla* has not undergone any recent genome duplication. **A** Blocks with more than 30 syntenic genes among *Amborella trichopoda*, *Vitis vinifera*, *Prunus mume*, *Betula platyphylla*, *Populus trichocarpa*, and *Arabidopsis thaliana*. Red lines show an example of one syntenic block in *Amborella* whose genome has not undergone gamma triplication matches up to three regions in *Vitis*, *Prunus*, and *Betula*, six regions in *Populus*, and 12 regions in *Arabidopsis*. **B** Pairwise *Ks* values of all homologous gene pairs from all synteny blocks in *the B. platyphylla* genome

To investigate the possible paleopolyploidization of the *B. platyphylla* genome, we performed all-against-all BLAST searches of the *B. platyphylla* genome with the *V. vinifera*^20^, *P. mume*^21^, *Populus trichocarpa*^19^, *Amborella trichopoda*^27^, and *A. thaliana*^22^ genomes. We chained the BLAST hits with a distance cutoff of 20 genes and a requirement of at least four gene pairs per synteny block using MCscan^28^ and QUOTA-ALIGN^29^. For each synteny region in the *A. trichopoda* genome, we found no more than three matches in the *Vitis vinifera*, *P. mume*, and *B. platyphylla* genomes. The results indicated that the most recent whole-genome duplication (WGD) in the *B. platyphylla* genome was eudicot hexaploidy, namely, “gamma duplication”^30^ (Fig. 2A). Therefore, the *Betula* genome, unlike those of *P. trichocarpa* and *A. thaliana*, has not undergone any recent WGD. The paleoploidy level of the *B. platyphylla* genome was further characterized by examining the *Ks* distribution of the duplicated gene pairs in the *B. platyphylla* synteny blocks (Fig. 2B). The *Ks* distribution in the *B. platyphylla* genome had a single peak corresponding to gamma duplication.

**Fig. 3:**
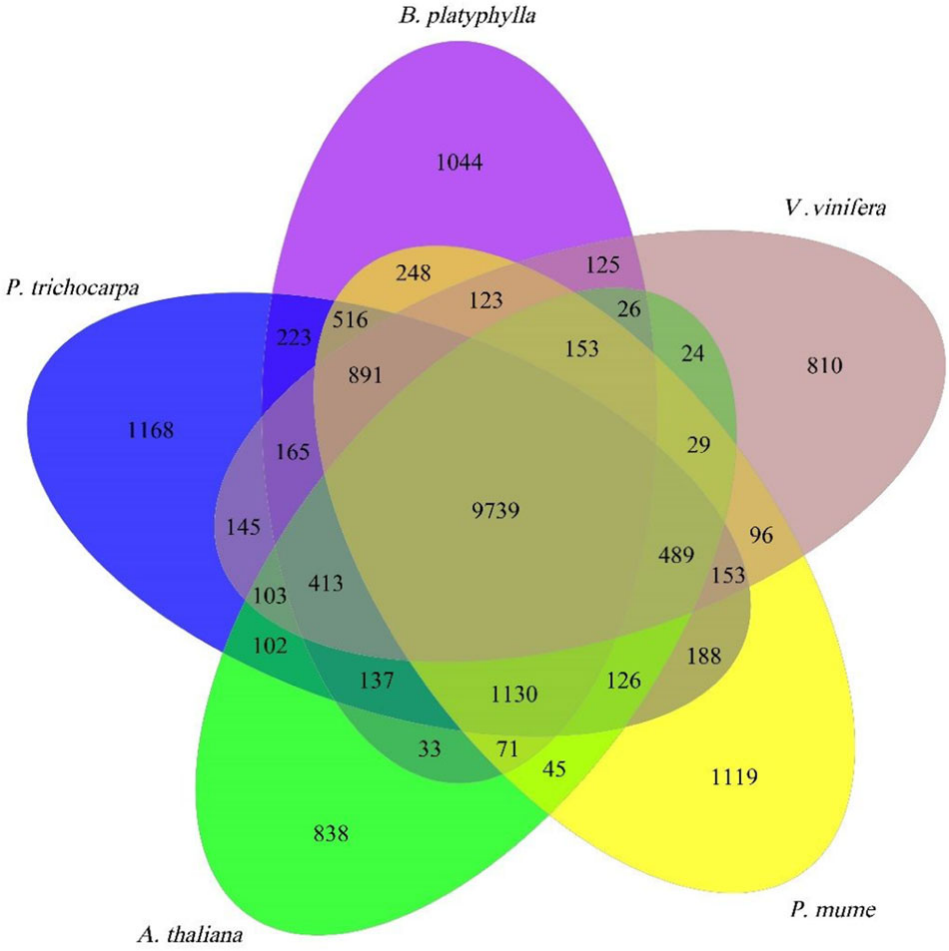
Venn diagram illustrating the shared and unique gene families among *Betula*, *Populus*, *Vitis*, *Arabidopsis*, and *Prunus*. The number over each region represents the number of genes belonging to that category

In addition to the phylogenetic analysis, we also performed a gene family analysis for the *B. platyphylla* genome. The comparison of gene families among *Betula*, *Populus*, *Arabidopsis*, *Vitis,* and *Prunus* revealed 9739 common gene families. In contrast, 1044 gene families are unique in the *B. platyphylla* genome (Fig. 3). A phylogenetic tree was built to analyze the divergence time of *B. platyphylla* from other species, including *B. pendula*. As a *Betula* species, *B. platyphylla* showed high similarity with *B. pendula*^31^. The two *Betula* species diverged approximately 2.6 million years ago. In comparison to *B. pendula*, 1254 gene families were expanded, while 667 gene families were contracted (Fig. S6).

### Wood formation

**Fig. 4:**
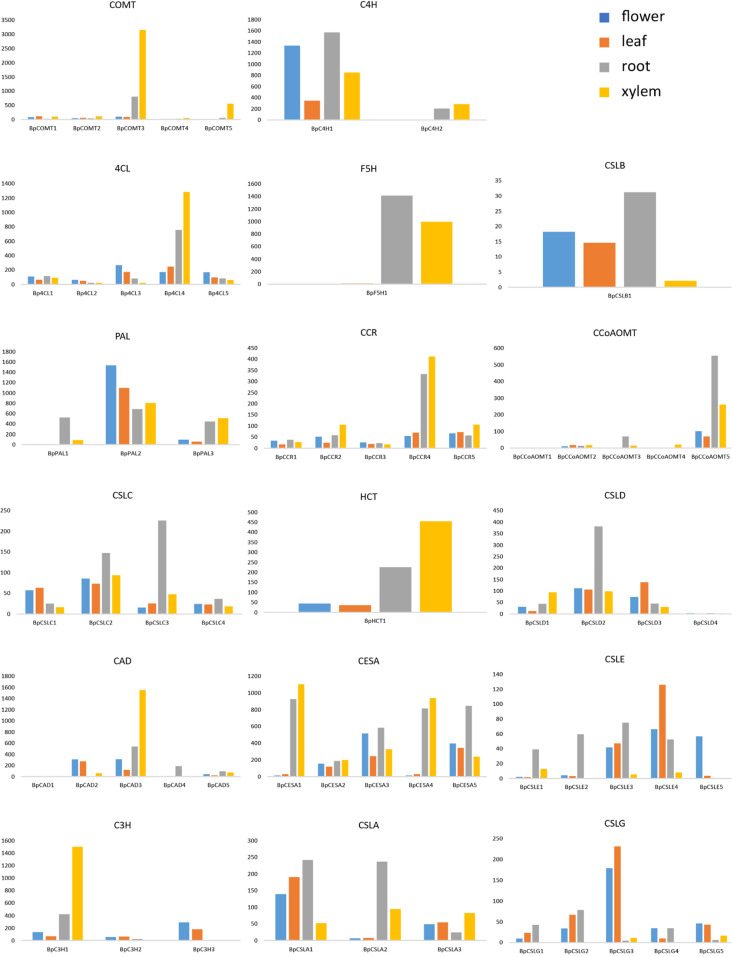
Tissue-specific expression of putative lignin and cellulose biosynthesis pathway genes identified through phylogenetic classification. No more than five of the most highly expressed genes are shown in roots (R), leaves (L), and xylem (X) if there are more than five homologous genes that encode the same enzyme. Expression levels were RPKMs generated from RNA-seq experiments on 3-year-old *B. platyphylla* trees grown in the field, and each was averaged from three replicates

*Betula* has evolved to possess a much higher syringyl to guaiacyl (S/G) ratio of lignin than many other woody species. A study of 11 species showed that *Betula* has an S/G ratio of 3.15, whereas *Populus*, *Acacia*, *Alnus*, *Acer*, *Liquidambar*, *Quercus*, and *Pinus* have S/G ratios between 1.18 and 1.76, and three *Eucalyptus* species have S/G ratios between 2.12 and 2.73 (ref. ^32^). Additionally, *Betula* has 19.6% Klason lignin, similar to *P. trichocarpa*, but this type of lignin is present at lower contents than in the other nine species (20.9–27%). A higher S/G ratio facilitates enzymatic hydrolysis of cellulose for renewable fuels^33^ and augments resistance to wood-decaying fungi^34^. We measured the S/G ratios in both CCR transgenic lines and wild-type plants of *B. platyphylla*; the S/G ratios we measured ranged from 4.09 to 5.27. To investigate the mechanisms responsible for the higher S/G lignin ratio, we identified 81 candidate lignin biosynthesis genes in the *B. platyphylla* genome. We examined the expression levels of these genes in the xylem, leaves, and roots of *B. platyphylla* and demonstrated tissue specificity of the following genes, *CCoAOMT* (*BpUN04756*), *CAD* (*Bp14G01282*), *4CL* (*BpUN04977*) and *CCR* (*BpUN05425*), *C3H* (*Bp14G00520*) and *HCT* (*BpUN05404*), which were especially expressed at higher levels in the xylem than in both roots and leaves (Fig. 4). Of these genes, the most highly expressed lignin biosynthesis gene in *B. platyphylla* was a caffeic acid 3-*O*-methyltransferase (COMT) gene (*BpUN00014*) in the xylem tissue (Fig. 4), which channels the intermediate products of the lignin pathway towards the biosynthesis of more syringyl lignin (S subunits). Correspondingly, the expression of coniferaldehyde 5-hydroxylase (*F5H*; *Bp5G00852*) in xylem appeared sufficiently high to ensure the biosynthesis of 5-hydroxy coniferaldehyde, which is the main precursor for COMT to synthesize syringyl lignin (Fig. 4). The high-level expression of these two genes aligns well with higher S/G in *B. platyphylla*. In addition to the putative lignin biosynthesis genes, we also identified putative genes implicated in cellulose synthesis, including 16 cellulose synthase (CESA) genes and 35 cellulose synthase-like (CSL) genes in the *B. platyphylla* genome (Table S4). We further analyzed gene families related to lignin biosynthesis in *B. platyphylla* and *B. pendula* (Table S5). We found that most of the gene families in the two species contain comparable members, such as the *PAL*, *C4H*, *HCT*, *C3H*, and *F5H* families. *B. platyphylla* contains more *CCoAOMT* and *CCR* members, while *B. pendula* contains more *4CL*, *COMT*, and *CAD* members.

### Cold response and tolerance

By virtue of its exceptional cold tolerance, *Betula* was an early colonizer of newly available lands following the most recent glaciation^35^. To better understand how *B. platyphylla* deals with cold stress at the molecular level, an RNA-seq time course experiment with six different time points was carried out. We identified differentially expressed genes (DEGs) between low-temperature conditions and the control at each time point. DEGs were judged by the thresholds of FDR ≤ 0.05 and |log2Ratio | ≥ 1. In total, we identified 2111, 3109, 3119, 3730, 3178, and 4384 DEGs after 6 h, 1, 2, 4, 7, and 14 days of low-temperature exposure, respectively. Among these DEGs, 879, 1294, 1244, 1585, 1342, and 1995 were upregulated, and 1232, 1815, 1875, 2145, 1836, and 2389 were downregulated at 6 h, 1, 2, 4, 7, and 14 days, respectively. We then identified unique DEGs for each time point and common DEGs throughout the whole process to dissect the dynamic biological processes of birch exposed to low temperature. Finally, 382, 347, 174, 550, 195, and 783 DEGs were specific to 6 h, 1, 2, 4, 7, and 14 days of low-temperature treatment, respectively. At the same time, 436 genes were identified as common DEGs. The unique DEGs may have specific functions and may be responsible for the physiological changes of birch during low-temperature treatment, and the common DEGs may participate in multiple biological processes and play important roles in the low-temperature response of birch.

We performed gene ontology (GO) enrichment to investigate the overrepresented biological processes involved in low-temperature stress. We first identified the enriched biological processes for DEGs at each time point. A total of 68, 95, 62, 72, 156, and 150 biological processes were significantly overrepresented based on FDR < 0.05 after 6 h, 1, 2, 4, 7, and 14 days of low-temperature exposure, respectively. We then built GO trees for these enriched biological processes to further understand the underlying molecular events. Interestingly, we found that response-related GO terms such as “response to stimulus, GO:0050896”; “response to stress, GO:0006950”; “response to abiotic stimulus, GO:0009628”; “response to temperature stimulus, GO:0009266”; and “response to cold, GO:0009409” were significantly enriched throughout the whole process. It is worth noting that “response to stimulus, GO:0050896” was the most enriched for all time points. In addition to the common enriched biological processes, some GO terms were enriched at specific time points. We found that three signaling biological processes, “cell surface receptor linked signaling pathway, GO:0007166”; “enzyme-linked receptor protein signaling pathway, GO:0007167”; and “transmembrane receptor protein tyrosine kinase signaling pathway, GO:0007169”, were significantly enriched at 6 h, 7 and 14 days. Three cell cycle-related GO terms, i.e., “cell cycle, GO:0007049, 2.30E−10”; “regulation of cell cycle, GO:0051726, 2.50E−08”; and “cell cycle phase, GO:0022403, 0.0099”, were enriched specifically at 7 days of low temperature exposure.

We also identified 184, 220, 195, 217, 242, and 341 differentially expressed transcription factors (TFs) of *B. platyphylla* after 6 h, 1, 2, 4, 7, and 14 days of exposure to low temperature. A total of 44 TFs were differentially expressed throughout the whole process of low-temperature treatment. It has been reported that the ERF family has important functions in the transcriptional regulation of biological processes related to the response to biotic stresses in various plant species. We found that 16, 23, 22, 21, 22, and 31 members of the ERF family were differentially expressed after 6 h, 1, 2, 4, 7, and 14 days of exposure to low temperature, respectively. In addition to the ERF family, many MYB, bHLH and NAC members were differentially expressed in birch when exposed to low temperature.

**Fig. 5:**
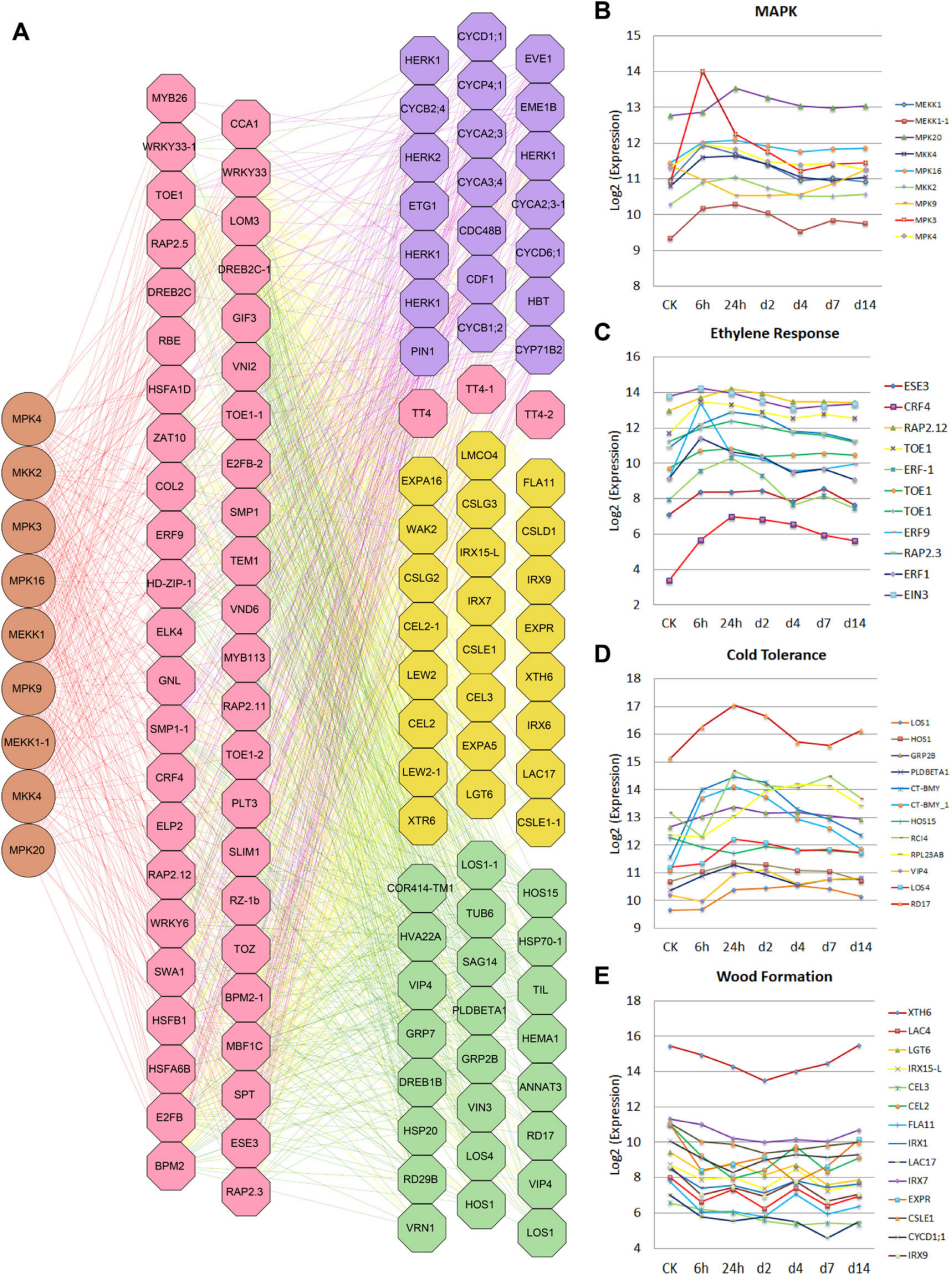
A three-layered gene regulatory network (GRN) was built using a bottom-up graphic Gaussian model (GGM) algorithm. **A** The top layer contains 9 MAPK genes, and the middle layer contains 58 transcription factors. In the bottom layers, genes involved in cold response and tolerance, wood formation, and cell division and growth were significantly up- or downregulated during cold treatment. **B**–**E** RNA-seq data were generated from the leaves of *B. platyphylla* in a cold-treated time-series experiment with six timepoints of 6 h, 12 h, 24 h, 2 day (48 h), 4 day (96 h), 7 day (168 h), and 14 day (336 h)

To better understand the gene regulatory relationships of the DEGs, we reconstructed the gene regulatory networks (GRNs) for the DEGs. Genes involved in the mitogen-activated protein kinase (MAPK) cascade (GO:0000165) were significantly upregulated at 6 h, as were the expression levels of genes involved in the ethylene-mediated signaling pathway (GO:0009873) and the osmosensory signaling pathway (GO:0007231). The genes involved in the cold response (GO:0009409) and cold acclimation (GO:0009631) were primarily upregulated at 24 h (Fig. 5). Using a gene association algorithm for ARACNE, we identified strong linear and nonlinear dependency between the genes present in the MAPK cascade and 58 TFs, which have dependency on 654 putative target genes. In *Arabidopsis*^36,37^, nine MAPK cascade genes were identified: MEKK1, MKK2, MPK4, MPK3, MKP1, MKK9, MPK16, MPK20, and MPK9. MEKK1, MKK2, and MPK4 are reported to function in a MEKK1–MKK2–MPK4 cascade in response to cold stress. In *B. platyphylla*, MPK3, MKP1, MKK9, MPK16, MPK20, and MPK9 are tightly coordinated with MEKK1–MKK2–MPK4 expression. We used a bottom-up graphic Gaussian model^33^ to build a three-layered GRN, with 654 putative target genes as the candidates for the bottom layer, 58 TFs as the candidates for the middle layer, and all 9 MAPK genes as the candidates for the top layer. The resulting network is shown in Fig. 5A. We found that 11 ethylene signal transduction genes and ethylene response factors were present in the middle layer, indicating that they, similar to their counterparts in other species^38^, play essential roles in cold response and acclimation. The majority of the genes involved in MAPK cascades were upregulated at 6 h (Fig. 5B), while most ethylene response genes (Fig. 5C) were significantly upregulated at either 6 or 24 h. Cold tolerance genes (Fig. 5D) were upregulated at 24 h and 2 days (48 h), decreased slightly at 4 days (96 h), possibly because of genetic reprogramming, and then remained relatively stable until the 14th day (336 h). In addition, some wood-formation genes, as shown in Fig. 5E, and some cell division and growth genes were significantly affected.

## Conclusions

This study presents the chromosomal-level *B. platyphylla* genome assembly, which has the same paleoploidy level as those of *V. vinifera* and *P. mume*. The most recent WGD in *B. platyphylla* is the core eudicot hexaploidization known as gamma duplication. Since then, the *B. platyphylla* genome has undergone significant rearrangements but no whole-genome duplication. The genetic basis underlying its noticeably high S/G ratio in wood lignin and extreme cold tolerance make this species a unique genetic and natural resource to investigate for specific utilization. The release of this *B. platyphylla* genome will lay the foundation for rapidly deepening our understanding of the evolution of the *Betula* genus, whose species have critical roles in many ecosystems in the Northern Hemisphere, from the boreal scrub to subtropical forests. The *B. platyphylla* genome and gene annotation provided here will enable accelerated genetic improvement of *Betula* for forestry by greatly facilitating marker-assisted breeding, genotype-phenotype studies, functional genomics, and genetic modification.

## Materials and methods

### Plant material and DNA sequencing

Genomic DNA was extracted from the roots of a 10-year-old *B. platyphylla* elite tree and sequenced using the PacBio and Illumina HiSeq4000 platforms. In total, we generated 20 and 34 Gb sequencing data using the Illumina and PacBio platforms, respectively. The obtained Illumina and PacBio reads corresponded to 45- and 77-fold coverages of the *B. platyphylla* genome, respectively.

### Genome assembly

Long-read sequencing yielded a total of 4.67 million reads with an N50 of 11.22 kb. The details of their sequencing statistical values are provided in Table S6. The PacBio reads were assembled into contigs using pb-assembly (v0.0.2)^7^ and wtdbg2 (v2.1)^8^. Assembly by pb-assembly was performed using the “--pa_daligner_option” option; the “e” value was set to 0.75, the “k” value was set to 18, and the minimum assembly length was set to 1000. The two sets of contigs were merged using quickmerge^9^ and then improved using FinisherSC (v2.1)^10^. The assembly result of pb-assembly was the query, and the assembly result of wtdbg was the database. The obtained contigs were polished using Racon (v1.3.1)^11^ and Pilon (v1.22)^12^ with the PacBio and Illumina reads, respectively. Racon and Pilon were run with default parameters. The organelle genomes were removed by mapping the contigs to the birch chloroplast and mitochondrial genomes using blastn (v2.7.1)^13^. The results were filtered by *E*-values < 1E−5 and a minimum match percentage of 80%.

### Anchoring the assembled scaffolds to genetic maps

A total of 250 F1 seedlings from the cross of *Betula platyphylla Suk* and *Betula pendula* var. carelica were used to construct the linkage map. We constructed a genetic map for *Betula* by employing specific-locus amplified fragment sequencing (SLAF-seq) technology^16^. The linkage map was constructed using JoinMap^39^. Finally, a genetic map including 14 linkage groups was obtained. Once the linkage map was determined, reads that contained SNP markers were aligned to the contigs using BWA-MEM^17^. ALLMAPS^18^ was used to anchor and orient the contigs to the genetic map.

### Identification of repetitive sequences in the *B. platyphylla* genome

Repetitive sequences were identified as follows: (1) Repetitive sequences with homology in RepBase (http://www.girinst.org/repbase) were identified using both RepeatMasker (http://www.repeatmasker.org) and RepeatProteinMask (http://www.repeatmasker.org). (2) We used RepeatModeler (http://www.repeatmasker.org), Piler^40^ and RepeatScount^41^ to perform de novo identification of repeats. (3) LTR retrotransposons were identified using LTR-FINDER^42^ with default parameters, and (4) miniature inverted-repeat transposable elements (MITEs) from the birch genome were identified de novo using the programs MITE-hunter^43^ and MITE Digger^44^.

### Gene prediction and annotation

Augustus^45^ and Genscan^46^ were used for de novo gene prediction, whereas TBLASTX^13^ was used for the identification of homologous open reading frames. Protein sequences of *Populus trichocarpa*, *T. cacao*, and *Arabidopsis thaliana* were used for homology-based gene prediction. We employed 116 RNA-seq data sets of *B. platyphylla* to identify and validate the gene models. GLEAN (http://sourceforge.net/projects/glean-gene/files/) was used to integrate the outputs of these methods. tRNAscan-SE^47^ was used to identify tRNA genes. INFERNAL^48^, with all RFAM models that previously generated hits from higher plants, was used to identify small nucleolar RNAs, snRNAs, microRNAs, and other noncoding RNAs. Gene functions were assigned according to the best hits in SwissProt (http://www.uniprot.org/) and TrEMBL (http://www.ebi.ac.uk/) using NCBI BLASTP^13^ (*E*-value ≤1e−5). We used InterProScan^49^ for the identification of protein domains. GO annotation for each gene was obtained from the corresponding InterPro entries.

### Synteny analysis

To compare the birch genome structure with other related plant genomes, we performed a synteny search. To call syntenic blocks, we performed all-against-all BLAST and chained the BLAST hits with a distance cutoff of 20 genes, with the requirement that at least four gene pairs were present in each syntenic block, using MCscan^28^ and QUOTA-ALIGN^29^. These criteria were used to extract syntenic anchors that were significantly clustered together based on previous studies^28,29^. The resulting dot plots were inspected to confirm the paleoploidy level of the *Betula platyphylla* genome in relation to other genomes. For Fig. 2A, we selected a set of syntenic relationships that show maximal retention so that the patterns are relatively unaffected by the post-WGD fractionation events.

### Gene family expansion or contraction

We constructed gene families in multiple species, including *Oryza sativa*, *P. mume*, *Theobroma cacao*, *A. thaliana*, *P. trichocarpa*, *V. vinifera*, *Eucalyptus grandis*, and the *B. platyphylla* genome assembly using OrthoMCL^50^. The analysis of changes in gene family sizes was conducted using CAFE^51^ software. The number of significantly expanded or contracted gene families was calculated for each branch (a species) in the phylogenetic tree. The enrichment analysis of GOs and protein domains was performed using genes in these expanded gene families of *B. platyphylla*.

### Identification of monolignol biosynthesis genes in *B. platyphylla*

We identified the monolignol biosynthesis genes in the *B. platyphylla* genome by reciprocal BLAST^13^ methods as described earlier^52^ using the 63 *A. thaliana* and 95 *P. trichocarpa* protein sequences of the ten known lignin biosynthesis gene families. These lignin biosynthesis genes were first searched against all *B. platyphylla* annotated gene models using BLASTP with a cutoff *E*-value of 1e−5. The resulting protein sequences of the *B. platyphylla* gene model hits were then queried against all proteins in the *A. thaliana* and *P. trichocarpa* genomes. The *B. platyphylla* gene models that had one of the *A. thaliana* or *P. trichocarpa* lignin genes as the top hit were subjected to phylogenetic analysis. Putative monolignol biosynthesis genes of *B. platyphylla* were then identified by selecting the closest birch homologous gene models to each of the 12 *A. thaliana*^53^ and 21 *P. trichocarpa* monolignol biosynthesis genes^52^.

### Generation of *BpCCR* transgenic lines

The *BpCCR1* transgenic lines used for the measurement of lignin were from a previous study^54^. Briefly, plant expression vectors of *BpCCR1* overexpression (35S::*BpCCR1*) and suppression (35S::anti-*BpCCR1*) were constructed using the Gateway System. The two constructs were transformed into birch genomes using *Agrobacterium*-mediated transformation. The young leaves of tissue-cultured plants were cut into small pieces, infected with *Agrobacterium* culture for 3–5 min, and then kept in the dark at 25 °C for 3–5 days. The leaves were then transferred onto selection medium and cultured for 2–3 weeks. The induced resistant calli were cultured on differentiation medium and then on budding medium for the induction of budding. When adventitious buds had sprouted to 3–5 cm shoots, they were cut and transferred into rooting medium for root induction.

### Generation of tissue-specific gene expression data

For tissue-specific expression analysis, we generated three RNA-seq sample datasets for each tissue (leaf, root, flower, and xylem) from 3-year-old *B. platyphylla* trees grown at the Experimental Station of Northeast Forestry University, Harbin, P.R. China. The tissues were harvested in the middle of July 2014, and there were three replicates for each tissue. The RPKM (reads per kilobase per million) normalized method was used to measure the expression levels in each tissue.

### Transcriptome analysis of *B. platyphylla* under cold stress conditions

An RNA-seq experiment with six time points (6, 24 h, 2, 4, 7, and 14 days) was designed to study signal transduction and the gene network in response to cold treatment. The cold treatments for different time points were initiated at different calculated times prior to the harvest time, and all treated plants were harvested at 10:00 a.m. on the same harvest day. Such a design was described earlier^55^. We performed two replicates for each treatment and three replicates for the control. The sampling materials for each replicate were pooled from the leaves of three independent plants of *B. platyphylla*. RNA extraction was performed using the methods described earlier in Zhang et al.^56^, and the RNA-seq experiment was performed at Novogene Bioinformatics Technology Co., Ltd. Raw sequence reads of each sample were obtained by aligning the clean reads to the *B. platyphylla* chromosomes obtained in this study. DEGs were identified using the edgeR package^57^ with a *P* value cutoff threshold of 0.05. A GO enrichment analysis of DEGs was performed by using our in-house pipeline, which was modified from Pop’s pipes, a software pipeline for analyzing *Populus* high-throughput gene expression data (http://sys.bio.mtu.edu). The gene expression matrix of *B. platyphylla* under low-temperature stress is listed in Table S7. A few significantly enriched biological processes that are relevant to signal transduction and cold response and tolerance are the MAPK cascade (GO:0000165), the ethylene-mediated signaling pathway (GO:0009873), the osmosensory signaling pathway (GO:0007231), cold response (GO:0009409) and cold acclimation (GO:0009631). To construct the MAPK-mediated GRN, we identified TFs with linear and nonlinear dependency on the nine MAPK genes and then the putative target genes that are dependent on these TFs using an efficient gene association algorithm called ARACNE^58^. We then used our bottom-up graphic Gaussian model algorithm^59^ to build a three-layered GRN, with putative target genes as the input for the bottom layer, TFs as the input for the middle layer, and all 9 MAPK genes as the input for the top layer. The full names of each gene used for the reconstruction of the GRN are listed in Table S8.

### Data access

The genome sequence data resulting from this birch genome project have been deposited in GenBank with the accession code PRJNA285437. The genome sequence and annotation will be submitted to Phytozome (https://phytozome.jgi.doe.gov/) and posted on our web portal for public use immediately after the manuscript is published (ftp://106.14.251.226/).

## Supplementary information

Supplementary figures

Supplementary tables
