# Supplementary material for: Genome sequence and evolution of Betula platyphylla
Source: Hortic Res. 2021 Feb 11;8:37. doi: 10.1038/s41438-021-00481-7 (PMC7878895; doi:10.1038/s41438-021-00481-7)
Supplement: Supplementary file 1 — Supplementary figures [file 41438_2021_481_MOESM1_ESM.doc]

**

**

**Figure S1. The global distribution of *Betula* species**.

**Figure S2. Sequencing coverage of the genome assembly**

**Figure S3. AT content of Miniature Inverted–Repeat Transposable Elements (MITEs) in the *Betula* genome.** AT of the majority of the MITEs in the *Betula* genome AT contents vary from 65% to 90*-% with the mode at 75%.

**Figure S4. Distribution of Miniature Inverted–Repeat Transposable Elements (MITEs) relative to genes in the *Betula* genome.** 44.9% and 40.5% MITEs prefer to insert into 5’ flanking regions (-1 ~ -5000 bp), and 3’ flanking regions (stop codon~5000 bp downstream), respectively. The remaining 14.6% MITEs were present in coding regions (including introns), which have an average length of 4,698 bp.


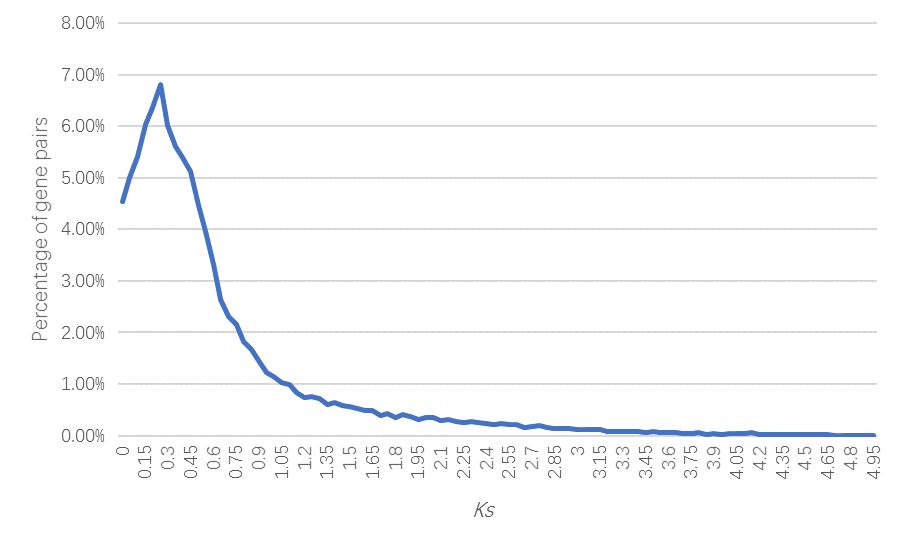


**Figure S5. Pairwise *Ks* values of gene pairs containing MITEs elements (750 bp upstream regions to 750 downstream regions).**

**
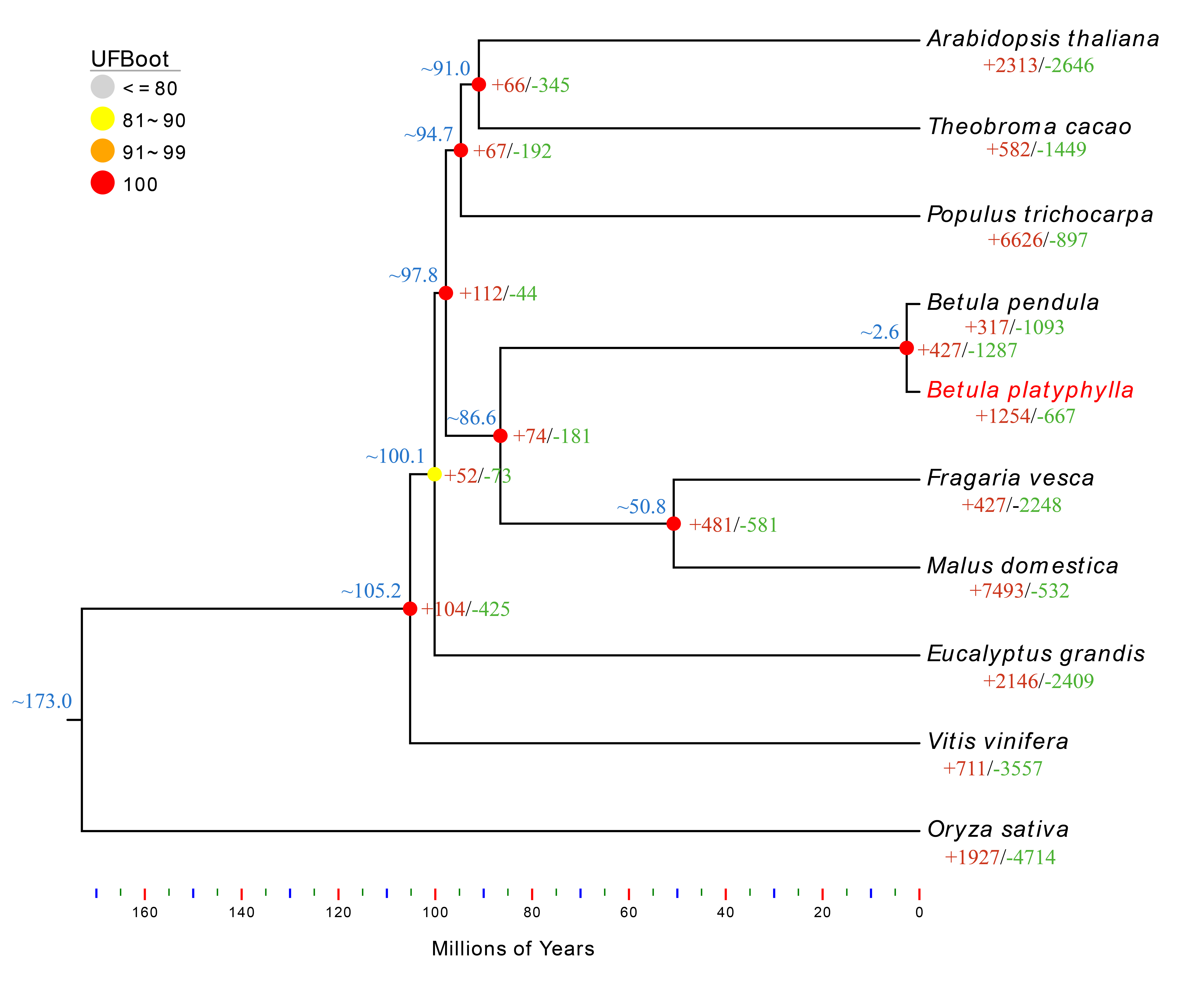
**

**Figure S6. Phylogenetic tree reconstruction of *Betula platyphylla* using maximum likelihood (ML) based on single copy homologous genes.**
